# Extreme Value Estimation Applied to Aerosol Size Distributions and Related Environmental Problems

**DOI:** 10.6028/jres.099.034

**Published:** 1994

**Authors:** Philip K. Hopke, Pentti Paatero

**Affiliations:** Department of Chemistry, Clarkson University, Potsdam, NY 13699-5810; Department of Physics, University of Helsinki, Helsinki, Finland

**Keywords:** aerosol mass concentrations, aerosol size distributions, deconvolution algorithms, maximally exposed individual, particulate matter 10 μm

## Abstract

This work examines the potential connections between extreme value statistics, problems in aerosol science, and a recent technique of solving ill-posed inversion problems, called EVE (Extreme Value Estimation). EVE estimates functional of the unknown solution by searching the extreme (maximum and minimum) values of that functional within a set of acceptable solutions. The statistics of occurrence of extreme values in real life were not considered when this method was developed. The results of this technique are more con servative than those of the other methods used to solve the problem of aerosol size distribution estimation like non-linear least squares, expectation-maximization, regularization, etc. The utilization of the customary methods of deconvolution may lead to an underestimation of the possibility of occurrence of extreme values in real life. It is suggested that consideration of extreme value statistics might aid in better defining the limits to be placed on the physically acceptable solutions in the EVE deconvolution. Other problems could also benefit from the application of extreme value statistics including the estimation of the second highest value of measured airborne particle mass in the context of the ambient air quality standard for particulate matter less than 10 μm and the determination of the Maximally Exposed Individual as required under the 1990 revisions to the Clean Air Act.

## 1. Introduction

Although extreme value statistics has been applied to environmental phenomena such as maximum wind speed and wave heights, it has not been applied to air pollution regulations, concentration estimation, or other related problems. Since many of the problems related to the effects of pollution on public health and welfare are dependent on the high end of the distribution of concentrations and/or exposures, there appears to be an opportunity to bring the developments in extreme value statistics to an area that could make good use of such methods. In this paper, three possible applications of extreme value statistics will be presented with the hope of sparking interest in bringing these tools to bear on some difficult but interesting problems.

## 2. Aerosol Size Distribution Estimation

One common problem in aerosol science is the estimation of the aerosol particles size distribution from measurements of their aerodynamic behavior (penetration or deposition) through a separation device. For small particles (< 300 nm), the penetration through a device is governed by the particle’s dtffusivity while for large particles (> 300 nm), inertial impaction is the usual separation mechanism. The response of the device is known either by calculation or measurement using particles of known size. For the unknown aerosol, the penetration is measured through a series of stages that sequentially remove additional particles. From the known characteristics and a limited number of measurements, the size distribution of the aerosol is estimated. In general there are fewer measurements than parameters to be estimated and there can be collinearity problems in the penetration matrix describing the instrument to complicate the problem further. There are a number of conventional approaches to providing a solution, but since the problem is underdetermined, one cannot insure that they will provide the true solution. It is also difficult to estimate error bounds for these solutions.

### 2.1 Conventional Methods

The observed sequence of particle concentrations penetrating through each stage of a size segregating device contains information on the size distribution of that aerosol. In general, the number of particles penetrating through a given stage of the system can be expressed by

$$N_{i}=N_{0}\int_{0}^{\infty }P\left(i,d_{p}\right)f\left(d_{p}\right)dd_{p}+\in_{i}$$ (1)

where *N_i_* is the concentration penetrating through the *i*th stage, *P*(*i,d*_p_) is the known particle size penetration characteristics for particles of diameter *d*_p_ through stage *i, f*(*d*_p_) is the size distribution function to be estimated, and *∊*_i_ is the error in fitting the measurement.

The normal approach to solving this equation is to express it as a series of linear, simultaneous equations relating the particle penetration fraction to discrete values of the size distribution and the stage penetration functions.

$$\begin{array}{cc} N_{i}=\sum_{j=1}^{I}P_{ij}\cdot f_{j} & \;i=1,...,I \end{array}$$ (2)

where *I* is the number of stages in the device, *J* is the number of size interval midpoints in the distribution, *P_ij_* is the penetration of the *j*th particle size, *d*_p_(*j*), through the *i*th stage, and *N_i_* is the number of particles penetrating the ith stage. The *f_i_* values must be nonnegative. However, there is generally no other objective *a priori* information on the nature of the distributions. The size distribution is not normalized so that

$$N_{0}=\sum_{j=1}^{J}f_{j},$$ (3)

where *N*_0_ is the total airborne concentration that is being partitioned into the various size intervals. Equation 2 can be rewritten in matrix form.

N=P⋅f+E. (4)

If *I* is greater than or equal to *J*, then the problem is overdetermined and can be solved for a unique solution using methods such as least squares. However, because the size distributions typically cover several orders of magnitude in particle diameter, it is normally necessary to estimate more midpoint values than measurements (*I* < *J*). There is then no unique solution to the problem.

Because collection by diffusion varies slowly with particle size, the penetration values for adjacent size ranges are often quite similar to one another. The penetration functions for a screen diffusion battery used for separating particles in the 0.5 nm to 500 nm range generally have substantial collinearity and thus, the problem is ill-conditioned as well as underdetermined [1]. Phillips [2] concluded that direct inversion of theses equations rarely produces physically acceptable solutions.

Two techniques for solving the ill-posed set of equations have been developed by Twomey [3] and by Maher and Laird [4]. There is limited theoretical justification for these methods. In practice, however, they have been widely used in the aerosol field with satisfactory results in many cases. Different variations of the Twomey algorithm have been proposed (e.g., [5]).

Other approaches have sought specific solutions within the feasible solution space by incorporating additional constraints on the problem. For example, Wolfenbarger and Seinfeld [6] assume that the distribution is fully smooth from one interval to another. However, it is certainly possible to have aerosol sources that produce particles with a very narrow initial distribution and thus, the overall aerosol size distribution may not be truly smooth. Thus, in all of these solution methods, a solution, but not necessarily the solution will be obtained.

### 2.2 Extreme Value Estimation

Replogle et al. [7] initially suggested the concept that the primary “solution” is the set of all those points that could produce the observed values. Paatero [8,9] recognized that this approach could be applied to the aerosol inversion problem by considering a one-to-many mapping of the measured *N* onto *f* such that there is the set *D*(*N*) of possible solutions corresponding to each possible measured *N*. The set *D*(*N*) is defined as the collection of all such solutions *f* that allow the reproduction of the measured *N* by Eq. (4) when reasonable values are used for *E*. Then the true unknown solution f is a member of the set *D*(*N*) with a high probability. *D*(*N*) is then the set of acceptable solutions. To initiate the analysis a best fit, *f*_0_, is calculated such that the nonnegative constraints are satisfied. Additional solutions are calculated that are sufficiently close to the best fit estimation that they fall within a criterion for acceptable solutions. For each of the estimated quantities, the largest and smallest values within the set *D*(*N*) are taken as the bounds of the confidence interval in which the true solution will fall at some high probability.

The question is then how to define what solutions are acceptable. The likelihood function, *L*(*N,f*) is the probability of observing *N* when f is given. It will be assumed that

$$-\ln\left(L\right)=const\cdot \sum_{i=1}^{l}{\left|\frac{E_{i}}{S_{i}}\right|}^{2}=const\cdot Q\left(f\right),$$ (5)

so that *Q*(*f*) is the sum-of-squares for the case in which *f* and *N* are substituted into Eq. (1). The optimum solution would then be the one that maximizes *L* or minimizes *Q*. The minimum *Q* value is denoted *Q*_0_ corresponding to *f*_0_. Maintaining the non-negativity constraints, the members of acceptable solution set, *D*, must be such that

$$\ln\left[L\left(f\right)\right]\geq \ln\left[L\left(f_{0}\right)\right]-const\cdot K$$ (6)

or alternatively,

$$Q\left(f\right)\leq Q_{0}+K,$$ (7)

where *K* is a confidence parameter with a typical value of 3. In this way, the set of acceptable solutions of the original equation that fit sufficiently well are determined. In estimating the effects of exposure to this airborne activity, it may be of interest to estimate a function of the distribution. The dose to cells in the bronchial epithelium could be calculated by

$$g\left[f\left(d_{p}\right)\right]=\int_{a}^{b}G\left(d_{p}\right)f\left(d_{p}\right)dd_{p},$$ (8)

where *G*(*d*_P_) is the dose per unit airborne alpha activity in the size range *d*_p_ to *d*_p_ + *dd*_p_ [10]. To examine the original distribution, the cumulative sums are estimated as represented by the following sequence of functionals:

$$\begin{array}{cc} F\left(d\right)=\overset{l}{\underset{j=1}{\Sigma }} & \begin{array}{c} \Delta_{j}=1\;\text{if}\;d_{p}\leq d\\ \Delta_{j}=0\;\text{if}\;d_{p}>d, \end{array} \end{array}$$ (9)

where the *F*(*d*) is the cumulative size distribution for the aerosol. The EVE(P) approach estimates such functionals by determining their confidence intervals.

### 2.3 Activity-Weighted Size Distributions

Activity-weighted size distribution have been measured in a number of normally occupied houses [11–13] using an automated, semi-continuous graded screen array (ASC-GSA) described by Ramamurthi [14] and Ramamurthi and Hopke [15]. The ASC-GSA measurement system is a diffusion battery that uses a combination of six sampler-detector units operated in parallel. Each sampler-detector unit couples wire screen penetration, filter collection, and activity detection with a solid state detector in a way as to minimize depositional losses. The system samples air simultaneously in all of the units, with a flow of about 15 lpm through the sampler slit between the detector and filter holder section in each unit. The sampled air is drawn through a filter. Complete details of the sampler are provided by Ramamurthi and Hopke [15].

Computer control of sampling, counting, and analysis permits automated, semi-continuous operation of the system with sampling every 1.5 h to 3 h. The activities of each radon progeny are estimated from alpha spectra collected during two counting intervals: the first one during sampling and the second 20 min after end of sampling. The observed concentrations of ^218^Po, ^214^Pb, and ^2l4^Bi are used to reconstruct the corresponding activity-weighted size distributions using the Expectation-Maximization algorithms [4] in six inferred size intervals in geometric progression within the 0.5 nm–500 nm size range. In addition to the individual size distribution for each decay product, the total airborne activity concentration can be characterized by the Potential Alpha Energy Concentration (PAEC). The *PAEC* can be calculated from the individual progeny concentrations by

$$\begin{array}{l} PAEC(mJm{)}^{-3}=5.79\times 10^{-7}\cdot c_{1}\\ +2.86\times 10^{-6}\cdot c_{2}+2.10\times 10^{-6}\cdot c_{3}, \end{array}$$ (10)

where *c*_1_, *c*_2_, and *c*_3_ are the activity concentrations of the three radon decay products in Bq m^−3^.

### 2.4 Results

**Fig. 1:**
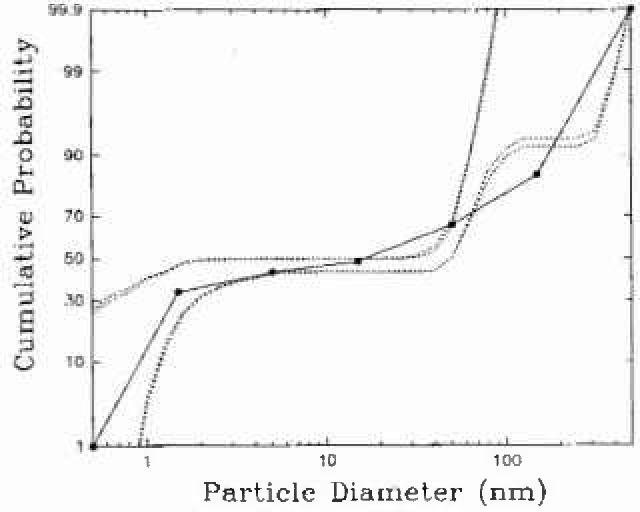
Cumulative distribution for PAEC for a sample taken in an occupied home in Arnprior, Ontario.

Measurements have been made in a number of houses in Northeastern North America. To illustrate the use of the EVE(P) algorithm for deconvoluting the activity size distributions, samples taken in houses in Arnprior, Ontario and Parishville, NY will be presented. In each home, radon and the size distributions of each of the three decay products and the PAEC were determined at 2 h intervals. The details of the experiments in Arnprior are given by Hopke et al. [11]. In this home, radon concentrations were relatively low (< 100 Bq m^−3^) and generally in the range of 25 Bq m^−3^ to 45 Bq m^−3^. The cumulative probability distribution for PAEC is shown in Fig. 1. The outer boundary lines are the EVE(P) results for the 95% and 99% confidence intervals. The solid central line is the EM deconvolu-tion result. Although the specific solution obtained by the EM algorithm should fall within the EVE bounds, it may lie anywhere within the feasible region. The confidence band will not necessarily be symmetrically distributed about the specific solution obtained by any particular algorithm.

**Fig. 2:**
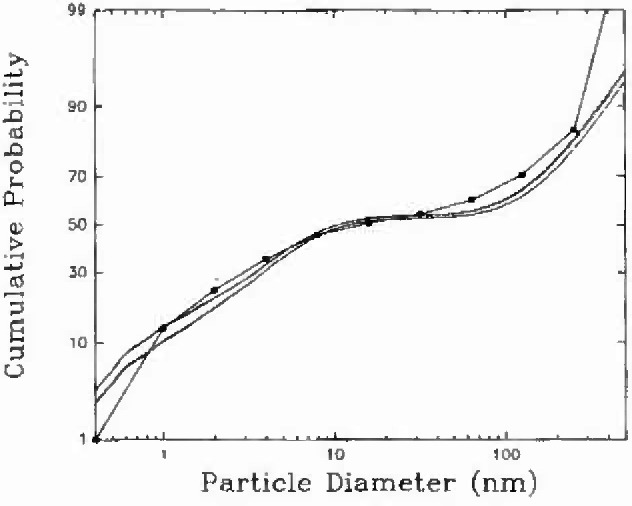
Cumulative distribution for PAEC for a sample taken in an occupied home in Parishville, NY.

Another analysis was performed on samples from a home in Parishville, NY with much higher radon concentrations (500 Bq m^−3^ to 600 Bq m^−3^) and thus, the bounds on the feasible region might be smaller [16]. The comparison of the EM size distribution with the EVE(P) distribution for PAEC is shown in Fig. 2. The EM-derived distribution does not appear to fully fit within the EVE(P) bounds. The question is then whether the current EVE(P) approach is the best description of the bounds on the feasible region.

Consideration of extreme value statistics could lead to the following suggestion: it might be possible to define some statistical properties for the extreme members of the set of acceptable solutions, even when there exists no general information about the probability distribution of the solution. Such properties might help in better defining the limits of the set of acceptable solutions. This could help in reducing the confidence intervals of the EVE decorivolution technique without sacrificing the reliability of estimation.

## 3. Other Applications

### 3.1 Ambient Air Quality Standard for PM_10_

In 1987, the U.S. Environmental Protection Agency promulgated a new National Ambient Air Quality Standard (NAAQS) for airborne particulate matter [17] which defined a size-selected portion of the ambient aerosol, particulate matter less than 10 μm or PM_10_, as important for protection human health and a new way of the determining when the standard had been violated. It is the form of the 24 h standard that involves extreme values. The standard requires that samples taken over 24 h intervals not show more than 1 “expected ex-ceedance” of 150 μg m^−3^ per year averaged over a 3 year period. Particle samples are not usually taken daily because of the manpower requirements needed to manually weigh unexposed filters, change them in the field, and weigh the exposed filters again. A minimum sampling regime would collect samples every 6th day. Thus, over a year approximately 61 samples might be collected. It is assumed that these samples are IID and thus, the number of “expected exceedances” can be estimated as

$$EE_{i}=OE_{i}\cdot \frac{n_{i}}{m_{j}},$$ (11)

where for a given year *i*, *EE_i_* is the number of estimated exceedances, *OE_i_* is the number of observed exceedances, *m_i_* is the number of samples taken, and *n_i_* is the number of days in the year. Thus, if 61 samples are taken in a 365 day year, then 1 observed exceedance becomes 6 expected exceedances. If this observed exceedance is the only one that occurs during a 3 year interval, then the 6 expected exceedances are divided by 3 years to yield an average number of expected exceedances of 2 which is greater than 1 and hence the area is in non-attainment of the standard. In other words, the average number of expected exceedances in any 3 year period is given by

$$E=\frac{1}{3}\cdot \sum_{i=1}^{3}EE_{i}.$$ (12)

Davidson and Hopke [18] examined some of the problems that arise as a result of the application of such a standard given incomplete sampling. To illustrate the difficulties, the upper tail of the distribution of airborne mass concentrations will be represented by the following exponential distribution:

$$P\left(c\leq L\right)=1-\frac{1}{365}=1-\exp(-y+2.0)$$ (13)

or

$$P\left(c>L\right)=\exp\left(2.0-7.90\frac{c}{L}\right),$$ (14)

where *c* is the mass concentration of airborne particulate matter and *L* is the maximum concentration allowable under the standard. The probability of an average number of exceedances being greater than 1 will be examined by examining *P*(*E* > 1.05).

$$\begin{array}{l} P\left(E\geq 1.05\right)=P(\sum EE_{i}/3\geq 1.05)\\ \;=P\left(\sum EE_{i}\;\geq 3.15\right)\\ \;=1-P\left(\sum EE_{i}<3.15\right)\\ \;=1-P\left(\sum OE_{i}<3.15\cdot n/m\right) \end{array}$$ (15)

Thus, the probability of nonattainment classification is dependent on the number of measurements per year.

$$\begin{array}{l} P\left(E\geq 1.05\right)=1-P\left(\sum OE_{i}=0\right)m\leq \frac{n}{3.15}\\ \;=1-P\left(\sum OE_{i}\leq 1\right)\frac{n}{3.15}<m\leq \frac{2n}{3.15}\\ \;=1-P\left(\sum OE_{i}\leq 2\right)\frac{2n}{3.15}<m\leq \frac{3n}{3.15}\\ \;=1-P\left(\sum OE_{i}\leq 3\right)\frac{3n}{3.15}<m\leq n \end{array}$$ (16)

The probabilities of observing 0 to 3 exceedances in any 1 year given the chosen sampling frequency can be estimated using the exponential distribution given in Eq. (17).

$$\begin{array}{l} P\left(\sum OE_{i}=0\right)={P_{0}}^{3}\\ P\left(\sum OE_{i}=1\right)=3{P_{0}}^{2}P_{0}\\ P\left(\sum OE_{i}=2\right)=3{P_{0}}^{2}P_{2}+3{P_{1}}^{2}P_{0}\\ P\left(\sum OE=3\right)={P_{1}}^{3}+3{P_{0}}^{2}P_{3}+6P_{0}P_{1}P_{2} \end{array}$$ (16)

**Fig. 3:**
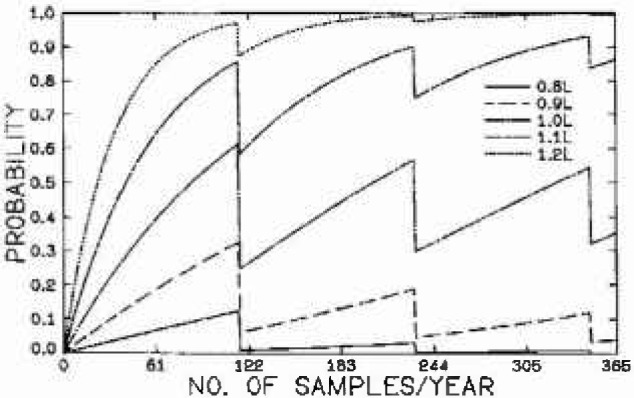
Probability of classifying an area as being in nonattainment of the 24 h NAAQS for PM_10_ based on an exponential distribution model of the tail.

A plot of the probability of declaring an area in nonattainment as a function of the number of samples taken per year is shown in Fig. 3. For *c* < 1.0*L*, classification as nonattainment is a Type I error. For *c* > 1.0*L*, probability of proper classification represents the power of the approach. The discontinuities occur because of the change in the integer values of the number of expected exceedances that occur at different n/m values. It can be seen that for an area that is exactly in attainment (*c* = 1.0*L*), there is a probability of up to 60% that it will be misclassified as nonattainment depending on the number of samples taken per year. This form of the standard, therefore, has a high probability of a type I error in order to attain a reasonable power to identify real nonattainment areas.

The goal of this standard is to have the second highest actual value whether measured or not, be at or below the prescribed concentration. Thus, alternative approaches that can more accurately estimate the second highest value in the tail of an extreme valued distribution would potentially provide equal or greater power white lowering the probability of making a misclassification error. Such an estimation process would make the standard more efficient while maintaining or possibly improving its effectiveness.

### 3.2 Most Exposed Individual

Under the Clean Air Act Amendment of 1990, the Congress has mandated that major emission sources of hazardous air pollutants, defined as materials on a list of 189 substances given in the Act, must install emission control systems. After these systems are in place, the residual risk to the *most exposed individual* must be assessed. If the risk is found to be > 10^−4^, the EPA Administrator must decide what additional steps, if any, are to be taken to reduce this risk. Previously the most exposed individual (MEI) has been defined as a person living continuously at the fence line of the facility 200 m from the emission source for 70 years. The idea of a 24 h per day, 70 year lifetime exposure for this individual is obviously an overestimate of the real maximally exposed individual. Recently EPA has revised its guidelines for exposure assessment to support the development of a distribution of exposures that an individual might encounter. However, extreme value statistics is never mentioned in any of the discussions of the use of the upper tail of the distribution to examine exposure and thus risk to the most exposed individual. Since the inaccurate estimation of the residual risk could result in substantial costs for no health benefit if the maximum exposure is overestimated or result in death or adverse health effects if underestimated, the best statistical methodologies should be applied to this important estimation problem. This situation appears ideally suited for extreme value statistics and thus should simultaneously provide interesting statistical problems to solve and value to the society by solving them properly.

## 4. Conclusions

There appear to be a number of areas in the air pollution field in which rigorous application of extreme value methods could provide useful contributions to solving important environmental problems. The better estimation of the bounds for aerosol size distributions, the determination of attainment or nonattainment of the NAAQS for PM_10_, and exposure and risk assessments at the high end of the range of possible exposures all could benefit from substantial involvement of extreme value statistical expertise. It is hoped that this report will spark interest in one or more of these problem areas.
